# A case report of small cell ovarian neuroendocrine carcinoma combined with immunochemotherapy

**DOI:** 10.1097/MD.0000000000031445

**Published:** 2022-11-18

**Authors:** Yuan-Xue Zhu, Xiang-Peng Gao, Lei Xin, You-Chao Jia

**Affiliations:** a Department of Medical Oncology, Affiliated Hospital of Hebei University, Hebei, P.R. China; b Hebei Key Laboratory of Cancer Radiotherapy and Chemotherapy, Baoding, Hebei, P.R. China; c Department of Medical Oncology, Affiliated Hospital of Hebei University, College of Clinical Medicine, Hebei University, Baoding, Hebei, P.R. China; d Key Laboratory of Precise Diagnosis and Treatment of Glioma, College of Clinical Medicine, Hebei University, Hebei Province, Hebei, P.R. China.

**Keywords:** case report, chemotherapy, immunotherapy, ovary, PD-1, small cell neuroendocrine carcinoma

## Abstract

Small cell ovarian neuroendocrine (NE) carcinoma is a rare NE tumor with a low incidence, poor prognosis, and no standardized treatment. To date, there have been no clear reports on the efficacy or prognosis of combined immunological and chemotherapy-based approaches in patients with this type of tumor.

**Methods:**

We administered the immune checkpoint inhibitor tirelizumab (PD-1 mab), in combination with etoposide and cisplatin chemotherapy (EP), to a patient with small cell ovarian NE carcinoma to examine its efficacy and safety.

**Results:**

The evaluation of efficacy was PR for every 2 courses of application, and immunomaintenance therapy was administered after 6 courses of treatment.

**Conclusion:**

Our studies indicate that tirelizumab combined with EP, may be an effective treatment for small cell ovarian NE carcinoma.

## 1. Introduction

Neuroendocrine (NE) tumors account for < 1% of all malignant tumors. These tumors can occur in the lungs, esophagus, stomach, pancreas, cervix, ovary, and multiple other sites. Gynecologic neuroendocrine carcinomas are even rarer, accounting for approximately 2% of all gynecological malignancies.^[1]^ Small-cell neuroendocrine carcinoma of the gynecologic tract is a pathological type of gynecologic neuroendocrine carcinomas, with incidence rates ranging from high to low as follows: small cell neuroendocrine carcinoma of the cervix, ovarian, endometrial, vaginal, and vulvar.^[2]^ Ovarian small-cell neuroendocrine carcinoma can be divided into 2 types: hypercalcemic small-cell carcinoma (SCCO of hypercalcemic type, SCCOHT) and pulmonary small-cell carcinoma (SCCO of pulmonary type, SCCOPT). SCCO has a relatively low incidence rate, yet a high degree of malignancy, rapid progression, high recurrence rate, and mortality, and there is currently no standardized treatment. Therefore, there is an urgent need for effective treatment to improve patient prognosis. The diagnosis and treatment of one case of small cell ovarian NE carcinoma admitted to our hospital are as follows.

## 2. Case report

**Figures 1-–7.**
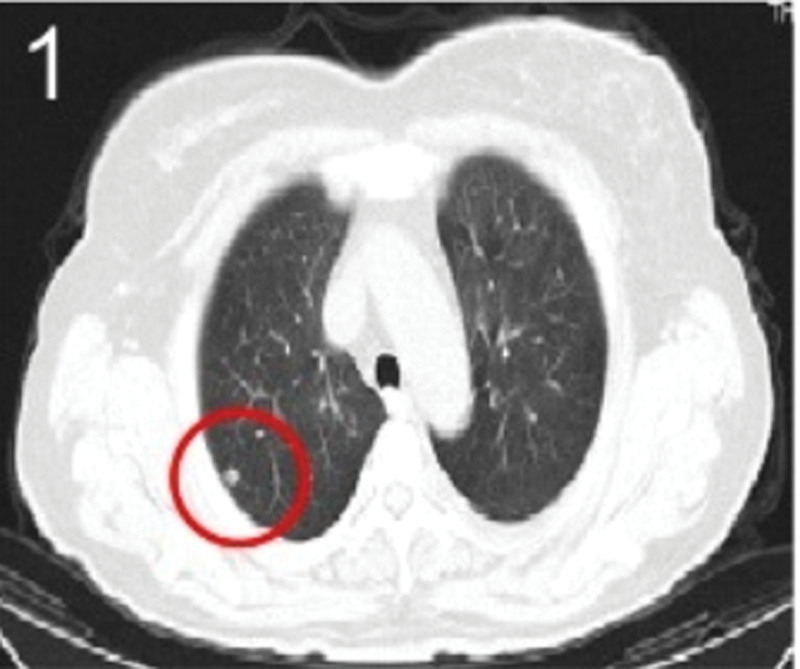
Shows the imaging results before treatment; 8–12 shows the reexamination images after treatment, and the red circle shows comparison of pulmonary nodules before and after treatment. (13 and 14) Cancer cells were closely arranged in sheets, with round or oval nuclei, fine granular chromatin, no obvious nucleoli, sparse cytoplasm, and unclear cell boundaries.

**Figure 2.**
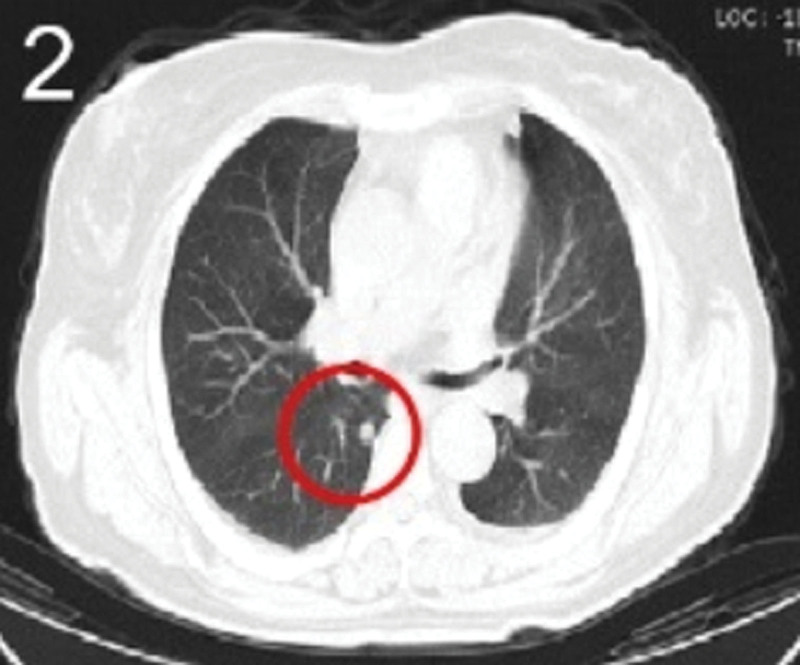


**Figure 3.**
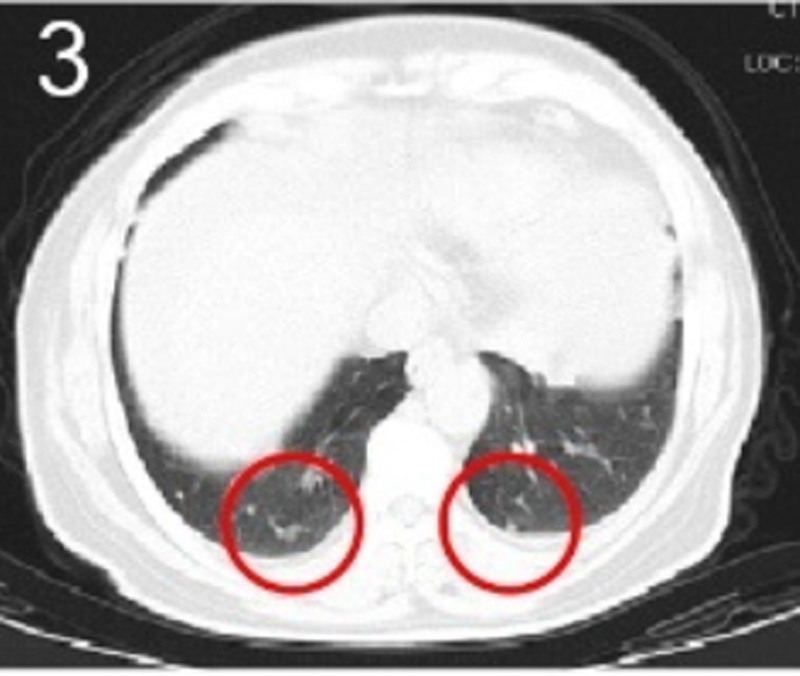


**Figure 4.**
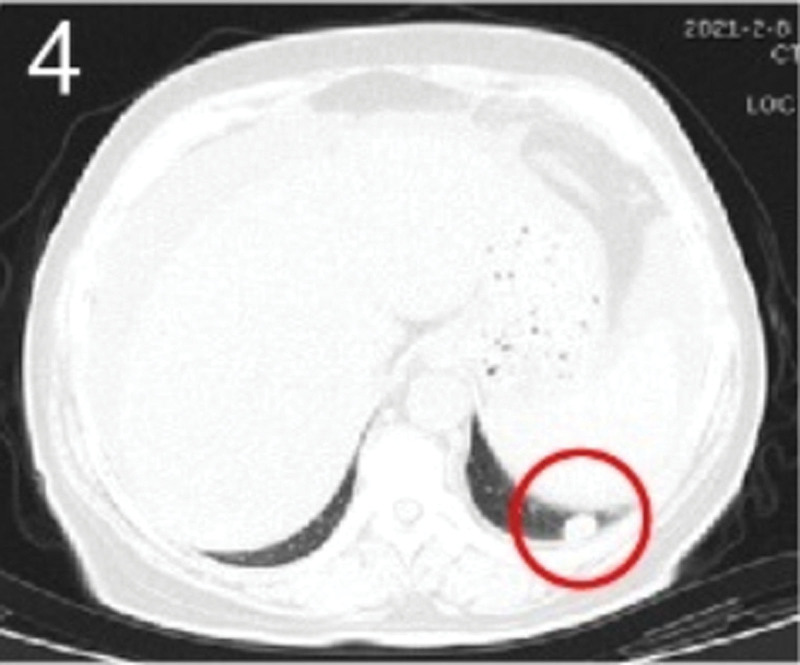


**Figure 5.**
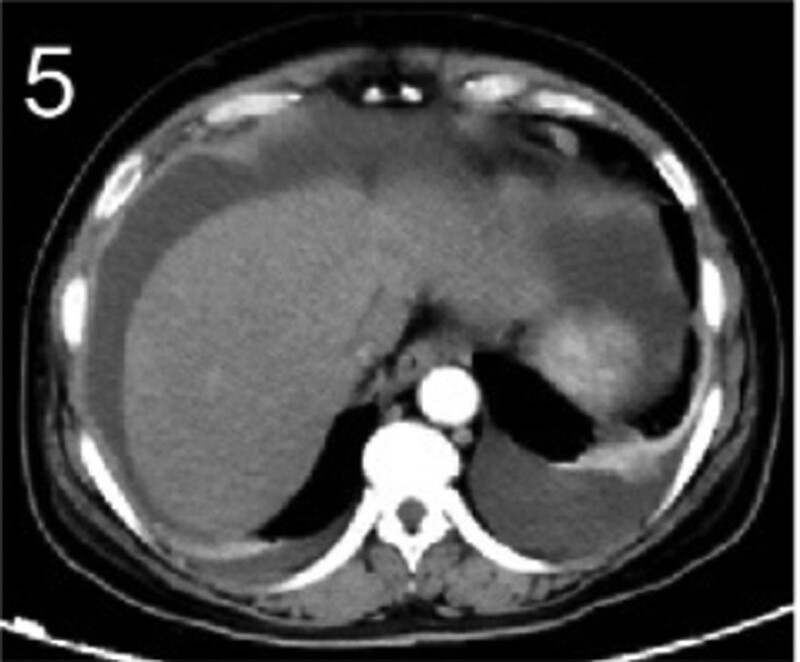


**Figure 6.**
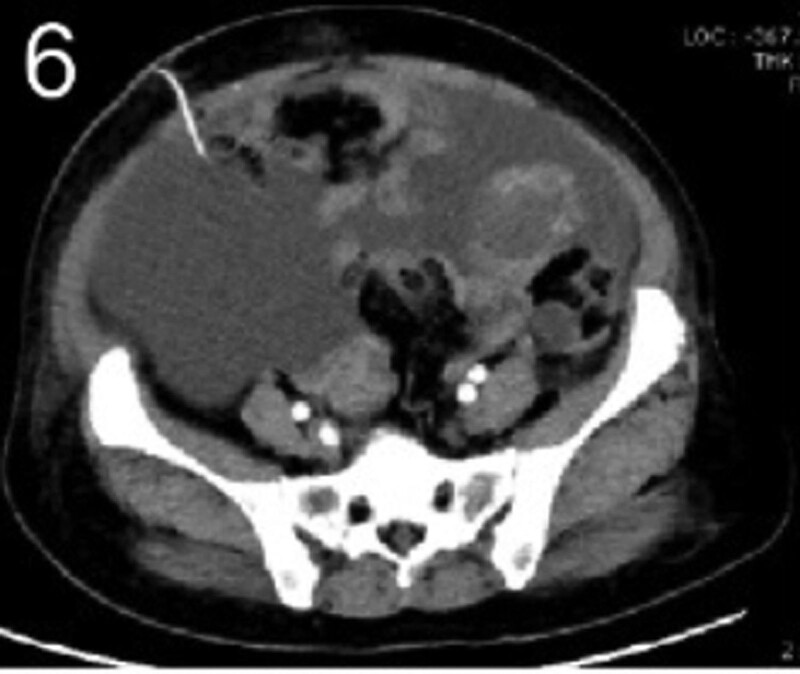


**Figure 7.**
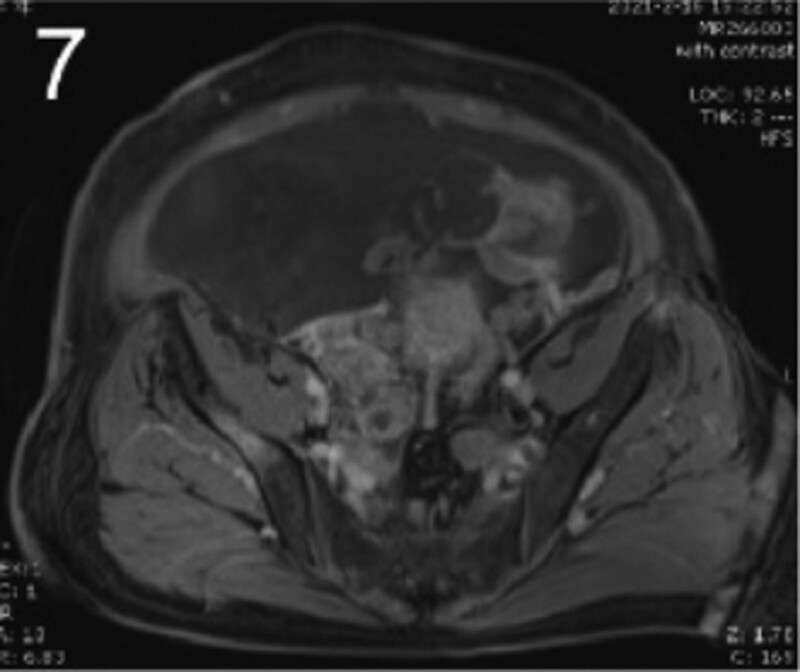


The patient was 55 years old, married, had 3 pregnancies and 2 births, and underwent natural menopause for 1 year. All medical history indicated that the patient was formerly in good health. She was admitted to the Department of Gynecology within the Affiliated Hospital of Hebei University in February 2021 due to “abdominal distension and hiccups that lasted for 7 days, and a bilateral adnexal mass had been present for 2 days.” Physical examination by a specialist concluded the following: abdominal conditions: abdominal distention was present, approximately the size of pregnancy at full term, the patient displayed abdominal tension, percussion dullness. Double diagnosis: married vulva, uterus and double attachment palpation is not clear, posterior uterus examination allows for touching an irregular hard mass the size of 4 × 3 cm, poor activity. Triad diagnosis: an irregular hard mass about 4 × 3 cm in size could be touched in the rectal fossa, which was immobile. The finger sleeve withdrew without blood stain. Auxiliary examination: CA125: 452.10 U/mL (0–35 U/mL). Combined transvaginal and transabdominal exploration ultrasonography: irregular low echo masses were found in both adjunct areas (the right side is about 7.1 × 4 × 3.5 cm, while the left side is about 6.9 × 6.9 × 4.4 cm, partly with unclear boundaries due to the bowel); Massive pelvic effusion was observed (almost 5.1 cm deep). Chest CT: multiple nodules in both lungs (Figs. 1–4); peritoneal effusion; abdominal enhanced CT showed thickening of the peritoneum, which was considered to be metastatic. Abdominal effusion (Figs. 5 and 6) and bilateral pleural effusion (Fig. 5) was observed. Pelvic MRI + enhancement showed bilateral adnexal area occupation, which is considered to be ovarian cancer (Fig. 7). Multiple soft tissue masses in the pelvic cavity were hypothesized to be metastatic. There were multiple abnormal signals in the sacrum, left femoral neck, bilateral ilium, and bilateral acetabulum, and metastasis was considered, as well. Pelvic effusion was observed in the patient. Peritoneal puncture biopsy and catheter drainage were performed on the patient. Ascites CA125 level was 771.3 U/mL. Laparoscopic exploration, biopsy, and a peritoneal thermal perfusion tube placement were performed under general anesthesia. There was an intestinal surface lesion that was believed to be a small cell malignant tumor. Print: tumor cells can be seen. According to the intraoperative exploration, FAGOTTI scores were 8, indicating that satisfactory tumor cell reduction could not be achieved. Pathological results of paraffin-embedded preparation of ascites cell sediment, combined with immunohistochemistry, allowed for the detection of tumor cells, which led to the diagnosis of NE carcinoma. However, some immunohistochemical indicators were not typical: Calretinin(−), CK7(−), Pax-8(−), WT-1(−), P53(+), P16(+), ER(−), PR(−), CA125(−), CK (weak+), P40 (−) and CEA(−), CD56(+), Syn(−), and NSE(−), Vimentin(scattered +), TTF-1(scattered +), CK20(−). Pathology of the lesion on the intestinal surface after biopsy concluded a small cell malignant tumor in the fibrous tissue (Figs. 13 and 14). Immunohistochemistry supported small cell NE carcinoma, with the results being as follows: CD56(+), CK(weak +), CK7(−), Ki67(80%+), LCA(−), P40(−), Syn(−), TTF-1(parts of +), CgA(−), P16 (+), Pax 8(−), and WT-1(−). The diagnosis was ovarian small cell NE carcinoma, pelvic lymph node metastasis, double lung metastasis, peritoneal metastasis, malignant peritoneal effusion, along with multiple bone metastases, indicating poor prognosis and short survival time. A single dose of cisplatin, administered through an intraperitoneal thermal infusion, at a dose of 110 mg was given.

**Figure 8.**
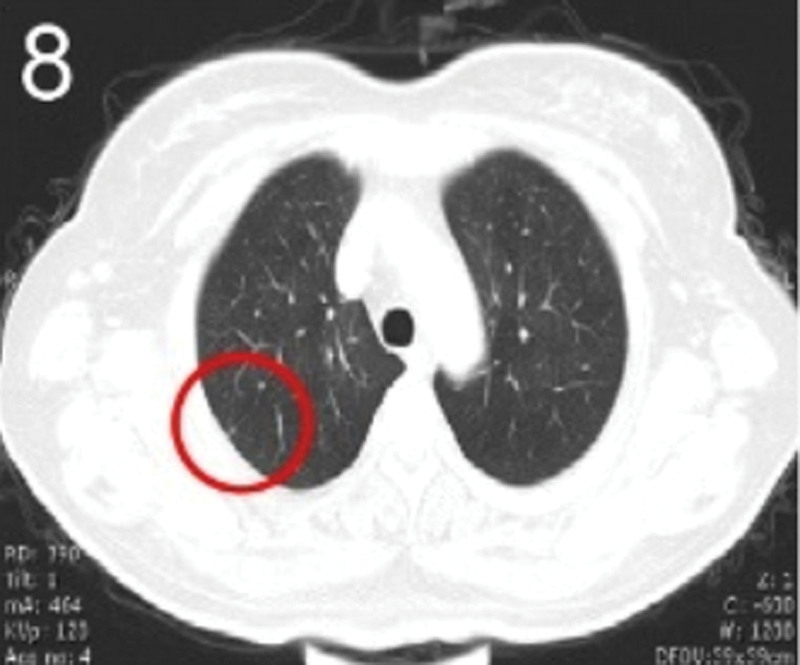


**Figure 9.**
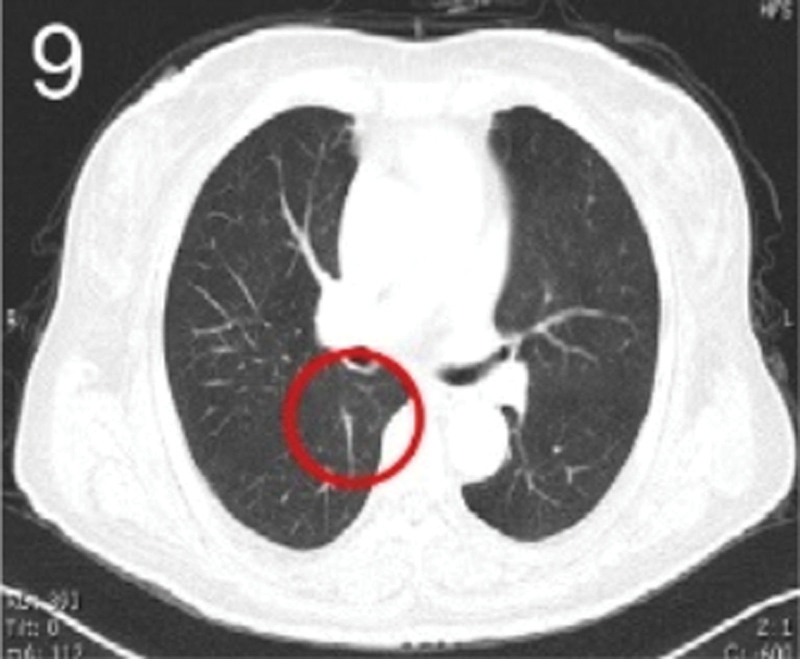


**Figure 10.**
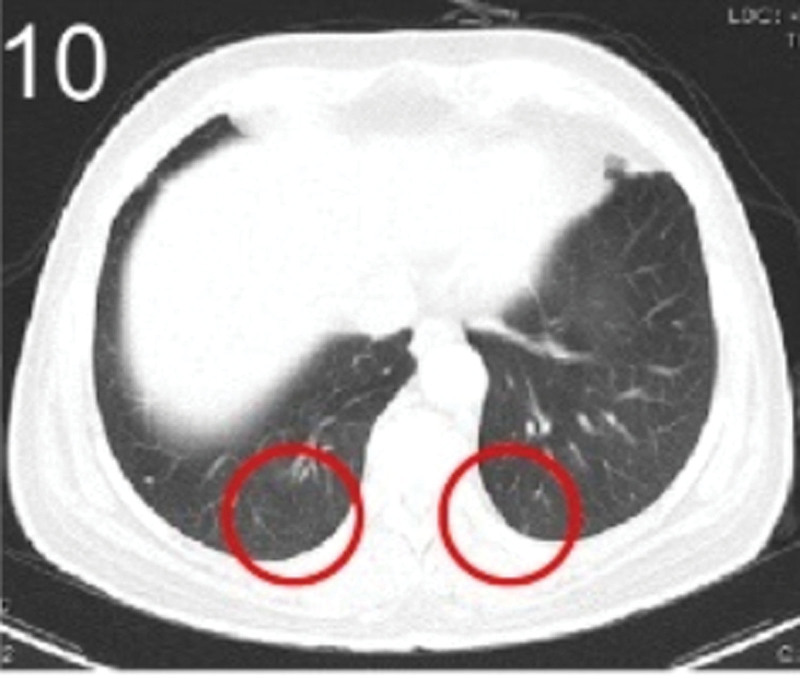


**Figure 11.**
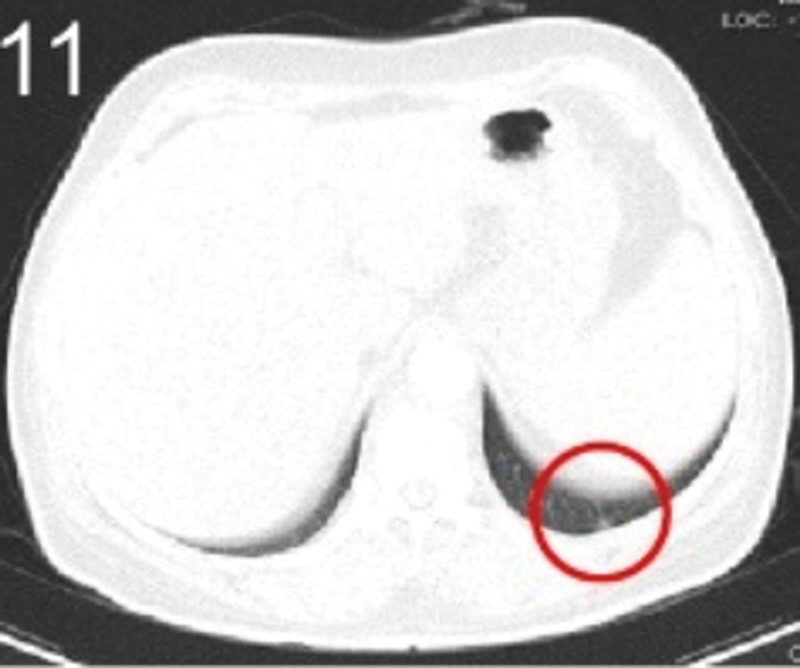


**Figure 12.**
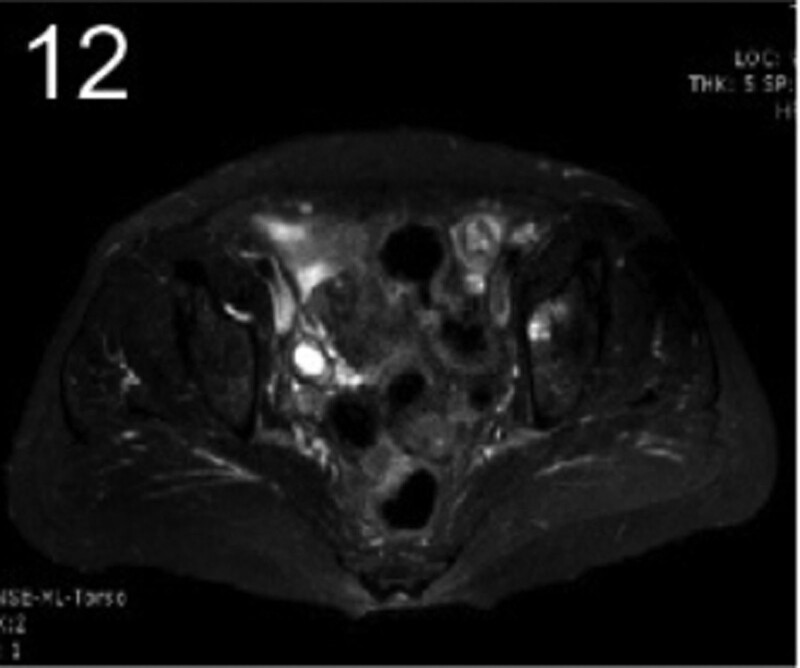


**Figure 13.**
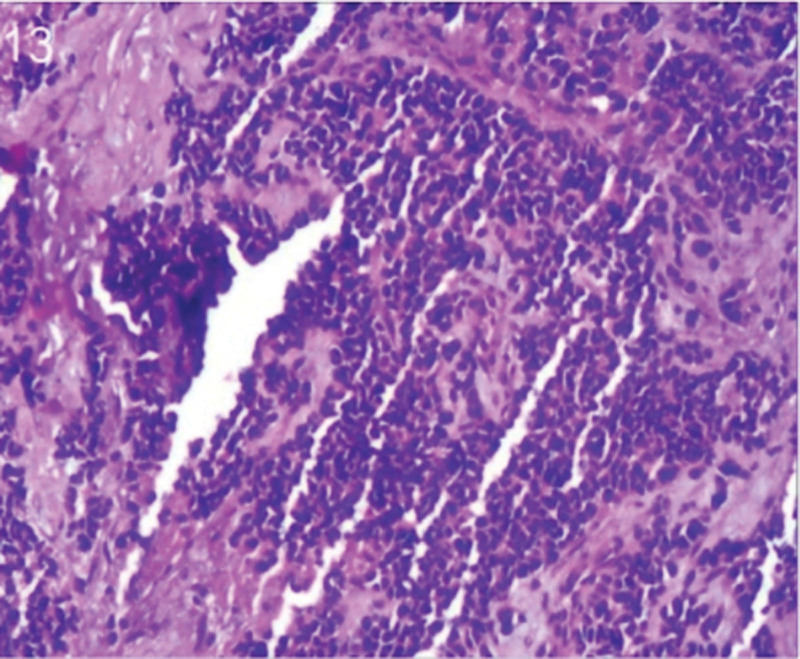


**Figure 14.**
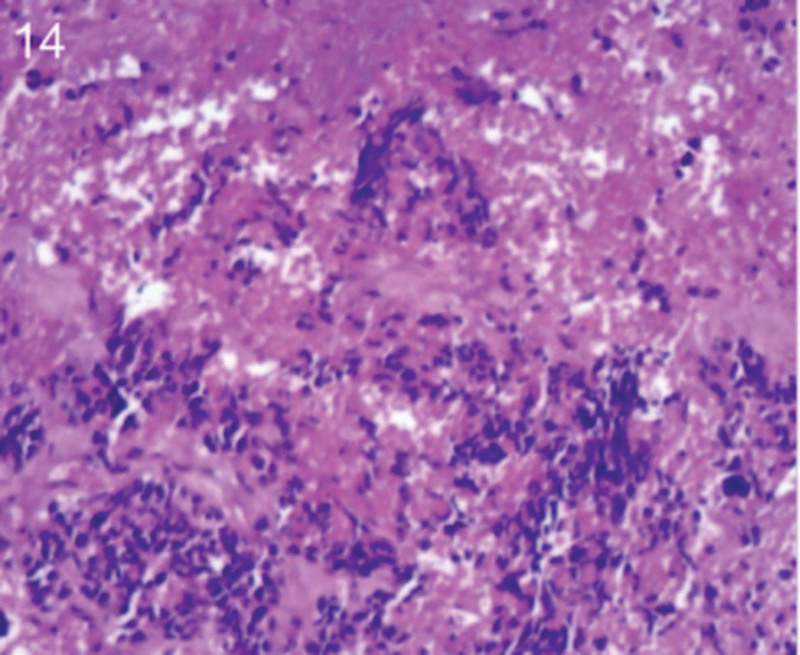


The patient was then transferred to the Medical Oncology Department, with PS at 2 points, CA125 levels of 193.2 U/mL. Ultrasonography showed bilateral pleural effusion, partial atelectasis, and peritoneal effusion. Six cycles of immunization combined with an etoposide and cisplatin chemotherapy (EP) regimen were administered from March 2021, and the administration schedule was as follows: tirelizumab at 200 mg on D0 IVGTT + etoposide at 100 mg on D1-4 IVGTT + cisplatin at 75 mg/m2 IVGTT, Q3W. Chemotherapy was reduced owing to intraperitoneal infusion of cisplatin in the first cycle. Before the second cycle, the patient’s abdominal distension and other symptoms disappeared, PS was 1, CA125 levels were normal, and NSE was 17.7 ng/mL. No treatment-related adverse events were observed throughout the regimen, except for grades 0–4 hematologic toxicity (neutropenia) and gastrointestinal symptoms (mild appetite loss and nausea). Two cycles later, enhanced chest and abdominal CT scans showed a significant reduction in the bilateral pulmonary nodules (Figs. 8–11), absorption of the left pleural effusion, disappearance of peritoneal thickening, and absorption of abdominal effusion. Additionally, pelvic MRI + enhancement showed that the bilateral adnexal area was significantly reduced (Fig. 12), the abdominal mass was not observed, and pelvic effusion disappeared. After 4 cycles of pelvic MRI + enhancement, PR was evaluated. After 6 cycles, comparisons of the pelvic MRI scans showed that the affected adnexal area continued to shrink, and the effect continued through the PR. After this, tirelizumab monotherapy immunomaintenance therapy was applied for 2 additional cycles, with the last application time being September 2021. Unfortunately, the patient displayed intermittent fever, loss of appetite, general fatigue, decreased muscle strength of the lower limbs, muscle pain, difficulty walking, and other symptoms outside the hospital. Blood tests revealed the following: AST 2013.8 U/L, creatinine 158 umol/L, HBDH 1668 U/L, LDH 2507.7 U/L, PLT 51 × 10^9^/L. Improved CT, bone marrow puncture, EMG, and other examinations were utilized to consider immune-related adverse reactions. This included assessments for immune-related hepatitis grade 4, immune-related nephritis grade 1, immune-related neurotoxicity (Guillain–Barre syndrome) grade 2, and immune-related hematotoxicity (immune thrombocytopenia) grade 2. Two mg/kg methylprednisolone sodium succinate shock therapy was administered for 7 days, along with liver and kidney protection, nerve nutrition, and other treatments. With these treatments, the patient’s symptoms were relieved, the medical indicators improved, and the hormone levels gradually reduced to discontinuation, followed by the complete withdrawal of immunotherapy. The last follow-up was conducted in April 2022. Liver and kidney function, myocardial enzymes, and platelets were all normal, the primary disease was considered stable. PFS has been ongoing for over a year.

## 3. Discussion

The incidence of lung small-cell carcinoma originating from the ovary is very low, and it is mostly observed in adult women with an average age of 59 years old.^[3]^ However, the disease progresses rapidly, with a high rate of malignancy and mortality. Another subtype is hypercalcemia, which is mostly observed in children and young women, with an average age of 23 years old. In this study, the patient was 55 years old, and serum calcium did not increase during hospitalization; therefore, this case was considered to be of the primary ovarian small-cell carcinoma-lung type.

The tissue origin of small cell ovarian NE carcinomas is uncertain. One theory suggests that this type of tumor may arise from stem cells or pluripotent cells within the surface epithelial cells that have NE and glandular differentiation capabilities. It is also theorized that this cancer may have been derived from non-neoplastic surface epithelial cells or NE cells in the neoplastic gonads.^[4]^ In addition, there are theories that ovarian NE small-cell carcinoma may have the same tissue occurrence as a primary carcinoid or a specific tumor entity.^[5]^ Cancer cells associated with small cell carcinoma of the ovarian lung type are small and round, with uniformly distributed nuclear chromatin and insignificant nucleoli. The tumor tissue contains epithelial–stromal tumor components and NE granules, which can be distinguished from ovarian metastasis in other small cell carcinoma tissues. NE cells are highly specialized neuro-like cells, and CD56 and SYN are the most sensitive NE markers. The diagnosis of small cell neuroendocrine carcinomas (SCNECs) depends on histological features, with IHC staining for NE markers being locally positive or even negative.^[6]^ Microscopically, the cancer cells in this patient were closely arranged in sheets with round or oval nuclei, fine granular chromatin, no obvious nucleoli, and a sparse cytoplasm. IHC staining of samples showed CD56(+), CgA(−), Syn(−), and NSE(−), which confirmed the previous statement. Differential diagnoses include metastatic small-cell carcinoma of the lung or elsewhere, hypercalcemia small-cell carcinoma of the ovary, carcinoid, primitive neuroectodermal tumor, and poorly differentiated stromal tumor.

There are few reports on small cell ovarian NE carcinoma in the previous literature, most of which were reported by individual cases or small samples without large sample size studies. Most patients have a poor prognosis after aggressive treatment and a high mortality rate within 2 years of diagnosis. Echhonr et al^[3]^ reported 11 cases of lung-type primary small cell ovarian carcinoma in 1992. Among the 7 patients who received long-term follow-up, 5 died or had accompanying diseases at 1−13 months post-treatment (mean, 8 months), one died after an unknown interval, and one was still alive at 7.5 years post-treatment. The other 2 had relapsed or residual disease at 6 and 8 months post-treatment. Among the 28 patients with small cell ovarian carcinoma reported in the literature, only 1 patient survived for 5 years without recurrence, while 4 patients survived with recurrence.^[7]^

In terms of treatment, there is no effective treatment for small cell ovarian NE carcinoma. Surgical resection remains the main treatment for early lesions, and most patients require chemotherapy or radiotherapy after tumor cell reduction. Bajaj et al^[8]^ reported that postoperative EP chemotherapy was associated with a significant survival advantage. Adjuvant chemotherapy provides a survival advantage for patients with or without lymph node metastasis. Neoadjuvant chemotherapy can also reduce the tumor to a certain extent and improve the radical resection rate; however, whether it can prolong the tumor-free time and overall survival of patients requires further study. For advanced or nonoperative patients, chemoradiotherapy is worth considering, although it has a higher recurrence rate and shorter survival.

With the rise of immunotherapy in recent years and encouraging results from other chemotherapy-resistant tumors, such as melanoma and kidney cancer, monoclonal antibodies are becoming more widely used in various types of cancer, showing increasing potential. Evidence suggests that the patient’s immune system plays an important role in the pathogenesis and treatment of NES, as immune cells can infiltrate the tumor microenvironment, leading to tumor-related inflammation.^[9]^ Immunotherapy is the most effective treatment for inflammatory tumor microenvironments. Patients with concurrent T lymphocyte infiltration and programmed cell death-ligand 1 (PD-L1) expression in the tumor microenvironment are likely to benefit from programmed cell death protein 1 (PD-1)/PD-L1 inhibition. Although NETs contain immunogenic epitopes that can be recognized by CD8 + T cells, there is still a lack of conclusive evidence that immune cells effectively recognize NET cells and develop immune memory.^[10]^ It has been reported that tumors with dMMR/microsatellite instability and high tumor mutation load exhibit more mutations and neoantigens, promoting tumor-infiltrating lymphocyte aggregation, which in turn leads to PD-1/PD-L1 overexpression.^[11]^ High levels of microsatellite instability have been demonstrated in gastrointestinal NECs, mixed adeno-NE carcinoma,^[12]^ and sporadic insulinoma^[13]^ but are rarely seen in other GEP-Nets.^[14]^ NECs have a higher mutation frequency than carcinoid tumors.^[15]^ All gynecological SCNECs have similar clinical behaviors and histopathological findings and are very similar to small cell lung cancer (SCLC), although they differ in etiology and risk factors. According to SCLC management and retrospective studies, the combination of ICI and cytotoxic chemotherapy theoretically becomes a treatment strategy with exploratory significance. In Impower-133,^[16]^ the median OS and PFS improved in the atezolizumab treatment group, and there was no significant difference in ORR when compared to the control group. In the CASPIAN test, median OS was prolonged, and ORR significantly improved in patients in the devaruzumab plus chemotherapy treatment group compared to those in the chemotherapy alone group.^[17]^ Survival data from these 2 trials suggest that chemotherapy, when combined with immunotherapy, is a successful treatment strategy for patients with extensive SCLC. The FDA has approved atezolizumab (anti-PD-L1 antibody), nivolumab (anti-PD-1 antibody), and pembrolizumab (anti-PD-1 antibody) for the treatment of patients with SCLC. The expression and immune response of PD-1/PD-L1 in gynecological NE carcinoma remains to be explored, and the benefits of immunotherapy remain controversial. In most cases, cervical NECs are associated with HPV and may develop high antigenicity, potentially becoming a target for immunotherapy. Paraghamian et al^[18]^ reported a patient with recurrent and metastatic cervical SCNEC whose PD-L1 expression was negative and who maintained CR for a long time after treatment with nivolumab was discontinued.

Small cell ovarian carcinoma has the microscopic and NE features of small cell lung carcinoma.^[3]^ Considering small cell NE carcinoma of the ovarian tumor treatment limitation, based on the stated earlier treatment strategies, we speculated that immunotherapy, combined with chemotherapy, is likely to be more effective in cases of small cell NE carcinoma ovary, but there is no clear relevant report to support this theory. The patient was treated with the PD-1 inhibitor tirelizumab, combined with the standard EP regimen, and PR continued after 2, 4, and 6 cycles of evaluation, followed by single-drug immunomaintenance therapy. The patient had PFS for over 1 year. However, due to the limited number of biopsy tissue samples, we could not obtain enough tissue for PD-1 and gene testing, and we could not determine the efficiency of combination and non-combination therapies. The number of cases of this disease was small and data support for large samples was lacking. Whether immunotherapy combined with chemotherapy can bring clinical benefits to patients is worthy of further multicenter and multi-regional clinical trials. Unfortunately, the patient subsequently developed severe multiorgan immune-related adverse reactions. The patient’s condition improved after treatment with a high dose of hormone shock, and immunotherapy was discontinued. Consequently, this resulted in failure to continue the study-related follow-up. At present, we still need to continue in-depth exploration and research, constantly summarize the experience in clinical work, be good at evidence-based innovation in the existing treatment standards and experience, further improve treatment strategies, improve efficiency, and closely monitor adverse reactions to improve treatment and patient survival.

## Acknowledgements

This study was supported by the Medical Science Research Project of the Hebei Province Health Department (grant no. ZD20140221).

## Author contributions

**Conceptualization:** Yuan-xue Zhu.

**Data curation:** Lei Xin.

**Formal analysis:** Xiang-peng Gao.

**Supervision:** You-chao Jia.

**Writing–original draft:** Xiang-peng Gao.

**Writing–review and editing:** Yuan-xue Zhu.

**Formal analysis:** Lei Xin.

**Funding acquisition:** You-chao Jia.

**Writing–editing:** You-chao Jia.

**Writing–original draft:** Yuan-xue Zhu, Xiang-peng Gao.
